# A Review on Mixed Matrix Membranes for Solvent Dehydration and Recovery Process

**DOI:** 10.3390/membranes11060441

**Published:** 2021-06-11

**Authors:** Priyanka Goyal, Subramanian Sundarrajan, Seeram Ramakrishna

**Affiliations:** 1 Birla Institute of Technology and Science-Pilani, Hyderabad Campus, Telangana 500078, India; pgpgoyal6@gmail.com; 2 Center for Nanofibers and Nanotechnology, Department of Mechanical Engineering, National University of Singapore, Blk E3 05-12, 2 Engineering Drive 3, Singapore 117581, Singapore; seeram@nus.edu.sg

**Keywords:** polydimethylsiloxane, metal-organic frameworks, zeolitic imidazolate frameworks, mixed matrix membrane, solvent separation, solvent dehydration, pervaporation

## Abstract

Solvent separation and dehydration are important operations for industries and laboratories. Processes such as distillation and extraction are not always effective and are energy-consuming. An alternate approach is offered by pervaporation, based on the solution-diffusion transport mechanism. Polymer-based membranes such as those made of Polydimethylsiloxane (PDMS) have offered good pervaporation performance. Attempts have been made to improve their performance by incorporating inorganic fillers into the PDMS matrix, in which metal-organic frameworks (MOFs) have proven to be the most efficient. Among the MOFs, Zeolitic imidazolate framework (ZIF) based membranes have shown an excellent performance, with high values for flux and separation factors. Various studies have been conducted, employing ZIF-PDMS membranes for pervaporation separation of mixtures such as aqueous-alcoholic solutions. This paper presents an extensive review of the pervaporation performance of ZIF-based mixed matrix membranes (MMMs), novel synthesis methods, filler modifications, factors affecting membrane performance as well as studies based on polymers other than PDMS for the membrane matrix. Some suggestions for future studies have also been provided, such as the use of biopolymers and self-healing membranes.

## 1. Introduction

**Figure 1:**
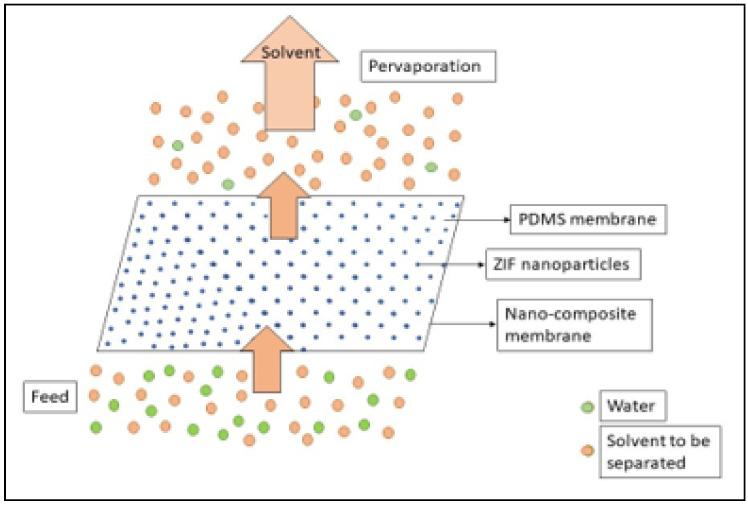
Schematic diagram of pervaporation performance.

Solvent dehydration and solvent recovery by pervaporation (PV) is attracting extensive attention as PV is a highly efficient and low energy-consuming process [1,2]. It is the process of the separation of dilute liquid mixtures through partial vaporization, with the driving force being the chemical potential across a membrane [3]. It is different from a simple vapour permeation as it involves the separation of liquid mixtures as opposed to gaseous mixtures. It is the combination of permeation and evaporation [4]. It follows the solution-diffusion transport mechanism [5] as shown in Figure 1, which describes mass transfer in three steps: (i) adsorption of target molecules on the feed side of the membrane depending on the chemical affinity of the molecule towards the membrane polymer, (ii) diffusion of the molecules into and across the membrane and (iii) desorption of the molecules on the permeate side [6]. This is based on the following principle- that component within a mixture preferentially permeates which has a higher product of solubility and diffusion rate [7]. The solution-diffusion mechanism involves the adsorption of the molecules on the membrane, which depends on the active functional groups, where the hydrophilic groups attach the water molecules and the hydrophobic groups attract the organic molecules. After the adsorption step, the molecules interact with the zeolite walls and diffuse across the membrane, following the two methods of configuration diffusion or molecular sieving which involves the separation based on molecule size, where small particles pass through the pores and the bigger ones are blocked by the pores. Finally, the molecules are de-adsorbed on the permeate side resulting from a concentration gradient [8] and the application of vacuum [9]. Intensive research is being conducted in the field to develop such membranes with excellent performance [5].

PV gives the advantage of superior separation efficiency as it does not depend on vapour-liquid equilibrium. In the case of azeotropic mixtures, it prevents cross-contamination that may occur due to a third component. As already mentioned, it consumes low energy as latent heat is only consumed by the penetrating component and temperatures involved are lower than those in other separation processes like distillation. To add to that, operational simplicity, flexibility, compatibility between different membrane operations in integrated systems and easy control and scale-up are few more advantages of this technology [7]. It makes use of no extra components and has a low cost of operation [10].

Studies have shown many other polymers to be potentially useful in the dehydration of solvents and solvent recovery. However, they have the limitation of commercial unavailability. For example, poly(vinyl alcohol) (PVA) has also been widely used, but it requires high activation energies [11] and has the problem of swelling which reduces the separation factor and selectivity [1]. Thus, based on previous research, PDMS has been considered as the state-of-the-art hydrophobic polymer to be used in making PV membranes due to its excellent performance and stability [4]. It shows good elastomeric and mechanical properties [12]. A ZIF/PDMS [13] membrane showed a better performance than ZIF/PEBA [14] membrane in terms of flux and separation factor values for the separation of butanol from an ABE fermentation broth under similar operating conditions. Similarly, other polymer membranes like PVA have also been used for pervaporation-based applications. Nonetheless, PDMS membranes have shown better performance with higher mechanical and thermal stability and long-term performance. However, the permeability of such membranes is lower than expected and considerable efforts have been invested in improving the permeability, one of them being the development of mixed matrix membranes (MMMs). These are made by dispersing nanostructured fillers into a polymer matrix [13], combining the lower costs, good compactness and ease of processability of polymers and high permeability of the porous fillers [5,15] (Table 1). Due to their pore structure, the porous fillers act as molecular sieves, while the non-porous ones create channels through the polymer matrix. The addition of fillers also helps disrupt the original polymer packing in the matrix, thereby, increasing the free volume and also the permeability [16]. Such MMMs have proved to be promising candidates for liquid as well as gas separation [5], with their characteristics like high selectivity and flux and long-term stability, even under different operating conditions [2].

MMMs are faced with the main issue of defect-formation during synthesis. This is mainly because of two reasons: (i) incompatibility between the polymer and filler and (ii) agglomeration of filler particles, causing the non-uniform distribution of the filler particles in the polymer matrix. Particle agglomeration can be seen through SEM micrographs [17]. These lead to interfacial defects which are of three types, namely interfacial voids, rigidification of the polymer chain layers and partial blocking of the pores [18]. The factors influencing the formation of the first two defects are weak adhesion between the polymer and the filler particles, the disordering of the polymer chains near the fillers, repulsive forces between the filler and the polymer, external stresses and different thermal expansion coefficients of the polymer and the fillers. Pore blocking is particular in the case of porous fillers and occurs when the pores are blocked by the polymer or the solvents from the separation mixture. The presence of rigid polymer chains can be verified by a change in the glass transition temperature of the MMM by using a DSC [17]. The presence of voids increases the permeability but decrease the selectivity of the membrane, while the reverse is observed for the rigid polymer chain layers. Blocked pores slow down the passage of the molecules through the membrane, thereby, affecting separation performance [19]. Few effective solutions have been proposed for this problem: (i) improving the interaction between filler particles and polymer, for instance, by electrostatic attraction, hydrogen bonding, and chemical bridging by using coupling agents [20], (ii) changing the method of the filler addition into the polymer matrix, for instance, by simultaneous spray self-assembly or interfacial synthesis and (iii) modifying the filler particles either physically or chemically [21], for instance, by the method of priming [18]. The dispersion of fillers was further improved by the filler suspension directly after filler preparation, i.e., before drying them in a PDMS solution. This in turn prevents aggregation and redispersion of filler nanoparticles after preparing the powder and which in turn showed better performance in biobutanol pervaporation [17].

The agglomeration of filler particles in the MMM membranes occurred when the prepared filler was dried and mixed with polymer and the agglomeration was avoided after the prepared filler suspension was mixed directly with polymer, which was also studied by TEM [17]. In the case of a suspension-dispersed filler-PDMS mixture, a more uniform degree of dispersion of filler nanoparticles was obtained, whereas poor dispersion was observed by TEM in the case of a powder dispersed filler/polymer matrix (MMM). This was due to the coating of polymer on the filler surface which thereby reduced the interphase voids in the MMM.

To develop MMMs that meet practical demands, MOFs have been used as the inorganic fillers due to their excellent attributes such as modifiable chemistry, in terms of structure and properties, as well as uniform pore size [5]. They have proved to be better fillers compared to other particles like zeolites or carbonaceous particles as they have better compatibility with the polymer due to their partial organic nature. Also, their hydrophobicity can be adjusted by selecting an appropriate ligand (for example, mesoporous silica spheres, see Table 1 for details) [22]. Among the MOFs, ZIFs are particularly investigated [23,24] as they offer easy procedures of synthesis and hydrothermal stability while operating [25]. Some of the other interesting properties of ZIFs are permanent porosities, high surface areas, adjustable pore sizes [7], high thermal and chemical stabilities [12]. They are the MOFs of transition metal ions (e.g., Zn^2+^) with imidazole organic linkers. In particular, ZIF-8 provides structural stability of up to 3 months in water and ZIF-7 and ZIF-90 are hydrophobic and show good hydrothermal stability [26].

**Figure 2:**
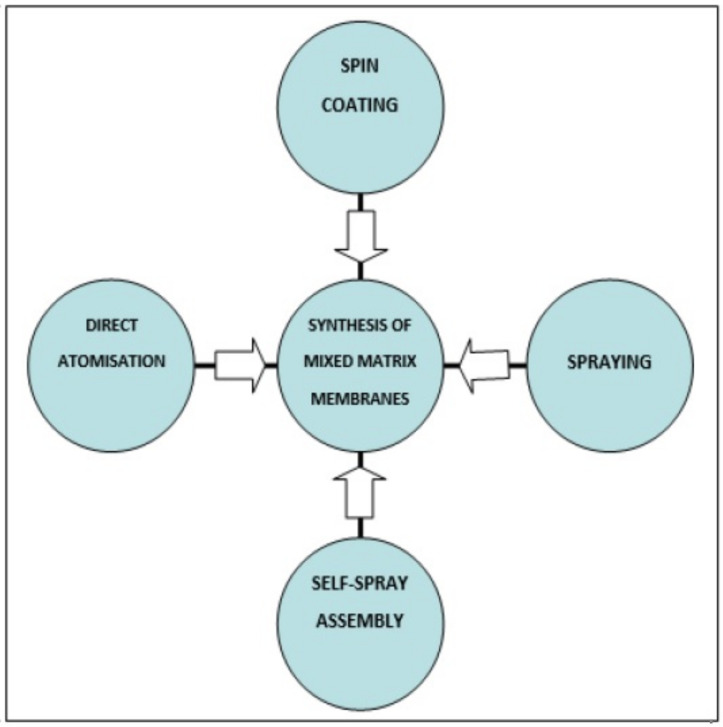
Different synthesis procedures that are used for MMMs.

In the case of synthesis, two broad categories of synthesis protocols proposed for MMMs are in-situ growth and secondary growth. In-situ growth occurs in one step where the support is immersed in the growth solution without attaching any crystals to the surface. Nucleation, growth, and intergrowth of ZIF all occur in the same fabrication step. Secondary seeded growth is a two-step procedure. In the first step, ZIF crystals are attached to the support by a seeding method followed by immersion into the growth solution [27]. Continuous thin ZIF-8 membranes on porous polymeric supports have also been prepared by a simple interfacial synthesis method, which can be easily upscaled [28]. There can be various synthesis procedures as shown in Figure 2. 

**Figure 3:**
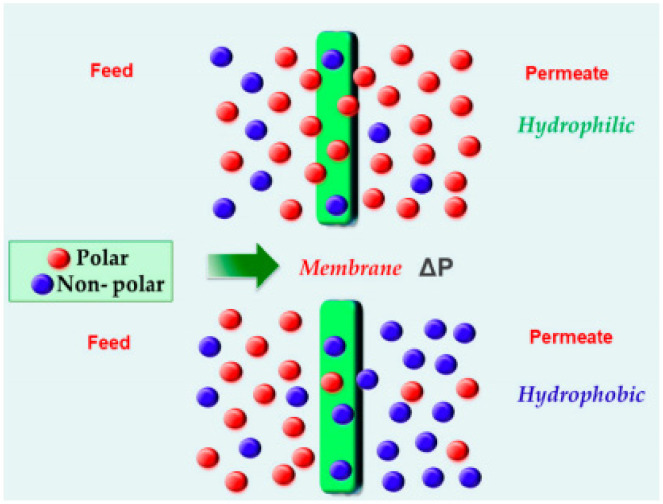
Preferential transport of hydrophilic and hydrophobic membranes. Adapted from [6] with permission from Copyright (2019), MDPI.

Membranes can be of two types depending upon the nature of the component to be separated: hydrophilic membranes that allow the preferential transport of polar molecules and hydrophobic membranes that allow the transport of the less polar or non-polar molecules, as shown in Figure 3 [6].

It has been reported that MMMs made of polymer matrix with ceramic fillers have also proven to be excellent in terms of gas separation. In a review conducted by Amooghin et al. [29], the performance of MMMs for CO_2_ separation has been comprehensively presented. Several other works involving the separation of hydrocarbon gaseous mixtures have been performed [30]. In this review, an extensive study on the pervaporation performance of ZIF based MMMS (in terms of flux and separation factor), novel synthesis methods, filler modifications, factors affecting membrane performance as well as studies based on polymers other than PDMS for the membrane matrix has been presented. A detailed discussion of membrane blending conditions, characteristics and separation performance has been presented in the form of tables for membranes based on PDMS as well as other polymers, including natural polymers. In addition, the use of biopolymers and self-healing membranes for pervaporation applications in future have also been suggested. 

### Solvent Dehydration and Solvent Recovery

When pervaporation is employed for the dehydration of a solvent, water is removed from the organic components by selective permeation through a hydrophilic membrane. Water is adsorbed onto the membrane on the feed side and is desorbed from the membrane on the permeate side. Due to the concentration gradient, the water molecules diffuse across the membrane and desorption in the form of water vapour occurs due to the application of the vacuum. For instance, a ZIF-8/PI membrane with PDMS coating was used for the dehydration of isopropanol [23]. Solvent dehydration is the most useful when a water-alcohol azeotrope mixture is to be dehydrated. Solvent recovery is also an important area where pervaporation is used. This is the opposite of solvent dehydration. In this case, the solvent molecules to be recovered are adsorbed on an organophilic membrane and diffuse towards the permeate side where they are desorbed as vapour and then recovered [9]. For instance, Xu et al. used a ZIF-90/PDMS membrane for the recovery of ethanol from an ethanol-water mixture [21]. 

## 2. ZIF/PDMS Membranes

### 2.1. Challenges in the Fabrication of MMMs

**Figure 4:**
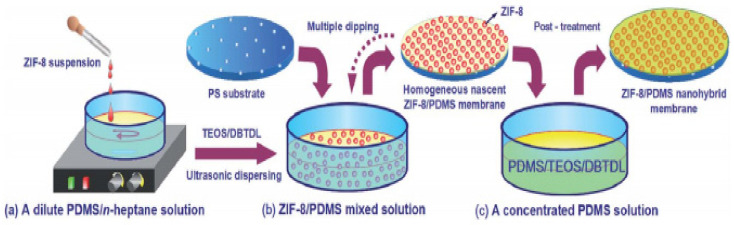
A novel synthesis method to avoid filler particle agglomeration [31]—(Republished with permission of Royal Society of Chemistry, from “Nanodisperse ZIF-8/PDMS hybrid membranes for biobutanol permselective pervaporation”, H. Fan, N. Wang, S. Ji, H. Yan, and G. Zhang, vol. 2, no. 48, Copyright (2021); permission conveyed through Copyright Clearance Center, Inc.).

Efforts have been made to overcome the issues faced by MMMs, one of them being nanoparticle agglomeration. Fan et al. [31] obtained a uniformly dispersed membrane, as shown in Figure 4, by repeatedly immersing a polysulfone support in dilute ZIF-8/PDMS suspension and subsequently immersing it in a concentrated PDMS solution without drying, thereby improving the dispersion of nanoparticles and removing the agglomeration defects. This membrane showed much higher performance in the separation of butanol with a flux of 2500.8 g/m^2^ h and a separation factor (SF) of 52.81.

One of the other challenges faced in the fabrication of MMMs is colloidal stability. To ensure better colloidal stability, a narrow particle size distribution as well as even dispersion of nanoparticles, Jin et al. [32] synthesised MMM using a drying free process. By avoiding the drying process, uniform and defect-free MMMs could be prepared as during the drying process, the Zn-imidazole groups on the surface typically reacted with each other and formed strong covalent Zn-Imidazole-Zn bonds, due to which the ZIF-8 nanofillers tend to agglomerate, which are very difficult to separate even upon subsequent re-dispersion. 

Fan et al. [33] reported a defect-free synthesis mechanism for MMMs involving a directly atomized oligomer (no solvent) and a nanoparticle-doped crosslinker solution while allowing the crosslinking of nanoparticles to occur on a rotating substrate surface. This process was also found to be time-saving when compared to traditional coating technologies. This technique was found to be universal, in the sense, that a range of nanomaterials could be dispersed in the polymer matrix, and the type of substrate could range from flat-sheet to hollow-fibre and tubular substrates. It was also found to be eco-friendly and produced stable membranes for biobutanol recovery with a flux of 2334.6 g/m^2^ h and a separation factor of 64.5.

Other challenging aspects of synthesising MMMs are controlling the grain boundary structure of the ZIF polycrystalline materials, which is generally considered as a defect and controls the nonselective inter-crystalline diffusion and the framework flexibility of the membrane. Due to this, the defect concentration varies for the membranes produced by the same synthesis route and so does their separation performance. A facile strategy to control these problems, as reported by Sheng et al. [25], was to subject the ZIF-8 polycrystalline membrane to a high pressure using a silicone rubber-PDMS coating. This resulted in an enhanced separation selectivity and separation factor by blocking the inter-crystalline defects and hindering the framework flexibility. It has been shown that although the performance factors, like flux and separation factors, were independent of the concentration of the PDMS solution used, they were dependant on the quality of the original ZIF membranes. Thus, the separation performance of a membrane is mainly affected by its constituent materials and structure. 

### 2.2. Superhydrophobic Membranes

The pervaporation performance of membranes are influenced by certain membrane surface properties like wettability and microstructure, and, hence, contact angle measurement for the membranes is conducted. It is well known that a more hydrophobic membrane surface is more repellent to water molecules, and thus excludes more water molecules. Only a few studies have been carried out on superhydrophobic membranes for pervaporation [34]. Some of the studies conducted on superhydrophobic membranes have been discussed briefly here.

**Figure 5:**
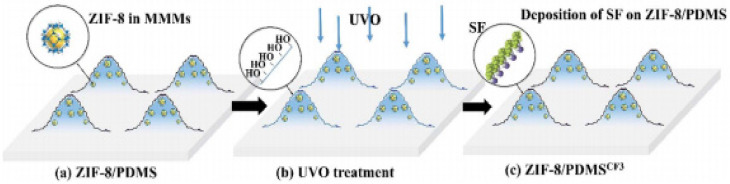
Synthesis of SAMs-modified ZIF-8/PDMS superhydrophobic membrane [34]—(Republished with permission of Royal Society of Chemistry, from “Designing superhydrophobic surfaces with SAM modification on hierarchical ZIF-8/polymer hybrid membranes for efficient bioalcohol pervaporation”, J. Li, N. Wang, H. Yan, S. Ji, and G. Zhang, vol. 4, no. 104, Copyright (2021); permission conveyed through Copyright Clearance Center, Inc.).

Several methods such as UV/ozone treatment [34], hierarchical micro and nanostructures [35], and modified fillers [36] have been applied to increase the hydrophobicity of the resulting membranes. The membrane obtained by Li et al. [34] was rendered superhydrophobic after UV/ozone (UVO) treatment and deposition of semifluorinated (SF) molecules, as self-assembled monolayers (SAMs) were formed on the hierarchical hybrid surface, as shown in Figure 5. SAMs were fabricated by the combinations of (i) creation of –OH groups and (ii) grafting reaction between SF trichlorosilane and –OH functionalities on the silica surface, which were created by the UVO treatment, in which hydroxyl groups were formed when atomic oxygen and ozone reacted with carbon atoms. These membranes had surface morphology with hierarchical structures similar to that of a lotus leaf, due to the ZIF nanoparticles. This enhanced the surface roughness, which amplified the hydrophobicity of the membranes.

For oil/water separation, surfaces with superhydrophobicity and underwater superoleophobicity have been greatly studied due to their high separation efficiency. Contributing further to the development of hierarchical micro and nanostructures on the membranes, Yuan et al. [35] presented a surface by a two-step designing of a unique 3-D multiscale ZIF-L on a 3D printed membrane. This approach involved the synthesis of two novel ZIF-Ls, the first obtained using an aqueous system with a relatively high concentration of 2-methylimidazole (Hmim) and zinc ions, displaying a three-dimensional leaf-crossed structure, and the second made by a secondary growth of small flat rod-shape and needle-like ZIF-Ls on the surface of leaf-crossed ZIF-L. These two ZIF-Ls were then deposited on a rough 3-D printed polyamide membrane in two steps. The hierarchical surface of the membrane made it hydrophobic. On being coated with PDMS, the membrane was endowed with extreme superhydrophobicity and superoleophilicity simultaneously. In a similar study conducted by Wang et al. [37], micro- and nanoscaled hierarchical structures were formed on the surface of a PDMS hybrid membrane. ZIF-8 nanocrystals combined with MCM-41_e formed the hybrid particles, which were having hierarchical architectures and were further modified by a hydrophobic silane coupling agent. This membrane was hydrophobic and showed good selectivity in alcohol permselective pervaporation.

Li et al. [36] too proposed a strategy to enhance the PV performance by increasing the hydrophobicity of the membranes by improving the hydrophobicity of the ZIF filler particles. First, the ZIF-8 particles were coated with polydopamine (PDA) which created a chemically reactive surface followed by modifying with silane coupling agents like propyltrimethoxysilane (PTMS) and octyltrimethoxysilane (OTMS). These membranes exhibited higher separation factors (47 with PTMS and 56 with OTMS) and slightly lower total fluxes (480.6 g/m^2^ h) than the usual ZIF-8/PDMS membranes, as well as higher mass transfer resistance for both water and butanol molecules while displaying an increased affinity for butanol and increased repellence for water molecules. As a result, although the butanol fluxes remained the same, the water fluxes showed a significant decline when compared to the membranes with unmodified ZIF. On comparing between the two modifiers also, the membrane with OTMS coupled ZIF particles (O-ZIF-8@PDA/PDMS) was characterized with a stronger hydrophobicity and lower porosity, resulting in a higher separation factor than the membrane with PTMS coupled ZIF particles (P-ZIF-8@PDA/PDMS).

### 2.3. Modified ZIF Particles

**Figure 6:**
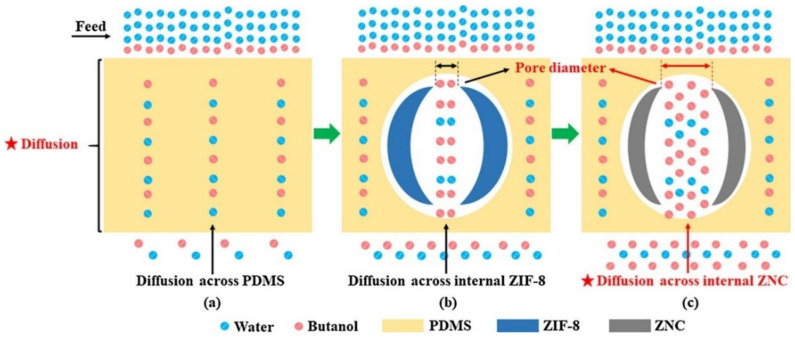
Illustration of the pure PDMS structure and the MMM and the main diffusion path of molecules. (**a**) The pure PDMS membrane; (**b**) The ZIF-8/PDMS MMM; (**c**) The ZNC/PDMS MMM [15]—(Reprinted from Separation and Purification Technology, vol. 221, Z. Si, D. Cai, S. Li, G. Li, Z. Wang, and P. Qin, “A high-efficiency diffusion process in carbonized ZIF-8 incorporated mixed matrix membrane for n-butanol recovery”, pp. 286–293, Copyright (2021), with permission from Elsevier).

Several modification procedures such as the introduction of carbons [13], microporous shells [22], ligand exchange methods [30] were applied to increase the structural stability of MMM membranes. Si et al. [13] examined the PV performance and stability of ZIF-8/PDMS MMM in an acetone-butanol-ethanol (ABE) fermentation broth. As per the expectation, the membrane displayed poor stability in ABE fermentation because of the presence of acidic by-products. This is because degradation of MOFs is accelerated in the presence of an acid, mainly caused as the proton and metal ion both compete for the coordinating linkers. As the MOF particles degrade, defects are formed in the dense layers, including interfacial gaps and collapse-induced voids, further deteriorating the membrane performance. As an alternative, ZIF derived nanoporous carbon (ZNC) were used as fillers. These particles were fabricated using direct carbonization and showed good acid resistance. ZNC/PDMS membranes were found to be advantageous in two more aspects: (i) they possessed a large pore diameter and pore volume that can significantly improve the diffusion process and (ii) there existed a good compatibility between ZNC and PDMS, resulting in the homogeneous dispersion of ZNC in the PDMS matrix, as shown in Figure 6. 

In another study by Naik et al. [22], ZIF was combined with mesoporous silica shells to include the benefits of both, a microporous shell, e.g., ZIFs, which have a high adsorption capacity, and a mesoporous core, e.g., mesoporous silica, which enhanced diffusion, resulting in improved separation capacity with fluxes of 1000 g/m^2^ h and 720 g/m^2^ h and separation factors of 13 and 15 for modified ZIF-71 and ZIF-8 respectively.

The result obtained by Yuan et al. [30] indicated that the 3-D framework of ZIF-8 was not damaged by shell ligand exchange reaction modification of 5,6-dimethylbenzimidazole (DMBIM), rather the amount of organic ligands increased which enhanced the interfacial compatibility between the fillers and the polymer matrix, leading to a homogenised dispersion of the ZIF-8-DMBIM nanoparticles in the PDMS matrix. A membrane with good structural stability was obtained as there were no defects found even after long-term operation. 

The use of ZIF-L nanosheets was another filler modification which produced membranes with excellent pervaporation performance, mechanical properties, thermal properties as well as long-term stability, attributing to the interfacial interactions (hydrogen bonding and van der Waal’s forces) between the ZIF-L and PDMS chains. These sheets possessed a unique leaf-like morphology, hydrophobicity and flexible pore structure, which allowed for preferential permeation of alcohols, resulting in high flux (402 g/m^2^ h) and separation factor (57.6) as shown in Table 1. The membrane selectivity was improved due to the tortuous and intricate pathways for water, which were rendered by the brick-and-mortar architecture. To prepare the membrane, the polymer was coated on its surface by the priming method [38].

A solvent assisted ligand exchange method was used to modify the ZIF-71 particles, using four different ligands- benzimidazole (BIM), methylbenzimidazole (MBIM), DMBIM and polyimide (PI). Membranes prepared by Yin et al. [39] using these ZIF-71 particles as fillers showed better selectivity but poor permeability for alcohol separation from aqueous solutions, as compared to unmodified filler particles. This was mainly because the modified particles had smaller pore sizes due to the larger sizes of these ligands as compared to the original dichloroimidazolate (dclm) ligand. Heterostructured fillers, such as ZIF-8 capped halloysite nanotubes, were embedded in the PDMs matrix to synthesize membranes with high permeability and selectivity for butanol pervaporation [40].

In a study by Zhu et al. [41], ZIF-8 particles were grown onto the surface of graphene oxide (GO) and the resultant ZIF-8@GO particles were used as fillers in the PDMS matrix for ethanol pervaporation. These modified particles showed good compatibility with PDMS and resulted in a uniformly dispersed membrane. With the synergistic effects of both, strong graphene oxide (GO) nanosheets and hydrophobic ZIF-8, the membrane exhibited improved performance. A strong compatibility was observed between the GO and ZIF-8 due to the strong hydrogen bonding and acid-base interaction between the carboxylic groups of the GO and the -NH groups of the ZIF-8, as well as the coordination bonding interaction between them. The optimal ZIF-8 loading on the GO nanosheets was found to be 87.5 wt%, as a lower loading led to higher hydrophilicity and a higher loading caused particle agglomeration, both resulting in reduced separation factors.

### 2.4. Modified Synthesis Procedures

**Figure 7:**
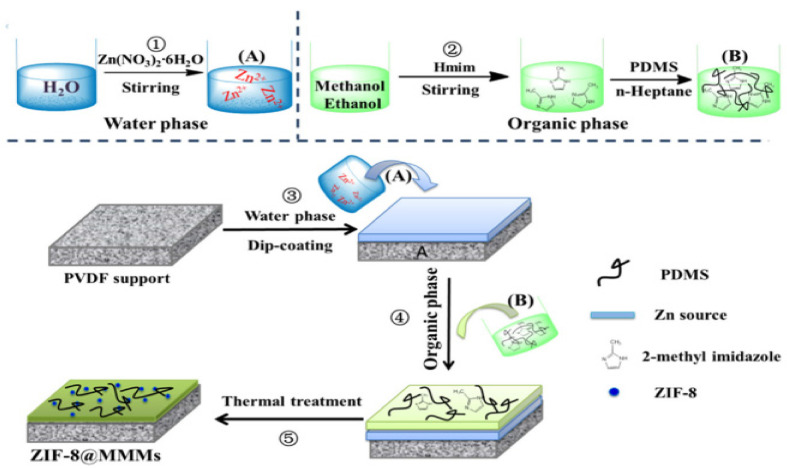
A novel preparation procedure. Preparation procedure of ZIF-8@MMMs membranes. (**A**) (in water phase) (**B**) (in organic phase) [42]—(Reprinted from Journal of Membrane Science, vol. 573, H. Mao, H. Zhen, A. Ahmad, A. Zhang and Z. Zhao, “In situ fabrication of MOF nanoparticles in PDMS membrane via interfacial synthesis for enhanced ethanol permselective pervaporation”, pp. 344–358, Copyright (2021), with permission from Elsevier).

Mao et al. [42] followed a novel approach towards the synthesis of ZIF/PDMS mixed matrix membranes as shown in Figure 7 below. These membranes gave an excellent performance, and their flux and separation factor values are given in Table 1. 

Zhao et al. [23] fabricated a well-intergrown ZIF-8 membrane on polyimide (PI) substrate with imidazole_2_-carboxaldehyde (ICA) as the covalent agent between the ZIF-8 layer and PI substrate, following a novel covalent-assisted seeding method. The PDMS surface coating mitigated the grain boundary of the ZIF-8/PI membrane, further improving performance. In the secondary seed growth method, the seed layer was pre-deposited on the substrate and the controlling factor was the adhesion between the two. Deposition of the seed layer could be done through dip coating, manual rubbing, microwave-associated method, etc. Another thin, dense, compact and hydrogen-selective ZIF-8 membrane was synthesized on a polymer/metal oxide (here, ZnO) mixed matrix support by secondary seeding method by Barankova et al. [27]. 

**Figure 8:**
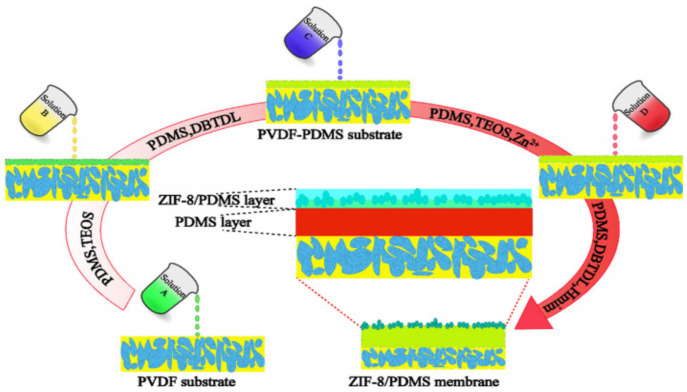
In-situ synthesis procedure [43]—(Reprinted from Separation and Purification Technology, vol. 236, G. Li, Z. Si, D. Cai, Z. Wang, P. Qin and T. Tan, “The in-situ synthesis of a high-flux ZIF-8/polydimethylsiloxane mixed matrix membrane for n-butanol pervaporation”, p. 116263, Copyright (2021), with permission from Elsevier).

Li et al. [43] fabricated a ZIF/PDMS MMM by adopting a novel in-situ synthesis (ISS) method via spin-coating, as shown in Figure 8. PDMS polymerization and ZIF-8 crystallization had occurred. An effective active layer was formed with a continuous and defect-free PDMS layer and a ZIF-8/PDMS layer, adding passable diffusion channels, overcoming the issue of physical incorporation of porous materials. 

A novel one-step synthesis technique was employed by Zhu et al. [44] to prepare MMMs doped with amine-functionalized ZIF-8. A strong covalent linking was observed among the PDMS, 3-glycidoxypropyltrimethoxysilane (GOPTS) and amine-functionalized ZIF-8 (AZIF-8), rendering a well-dispersed membrane with excellent polymer-filler compatibility, eliminating the interfacial defects. Such membranes showed higher flux and separation factors when compared to non-modified filler doped MMM. 

The above-modified preparation procedures such as interfacial synthesis, covalent assisted seeding, in-situ synthesis of fillers in a polymer matrix, and covalent linking of PDMS and functionalized fillers are some of the effective ways to overcome grain boundary or interfacial defects between filler and polymers and to achieve a higher separation performance.

### 2.5. Modelling Studies on MMMs

To study the structural and diffusive properties of ZIF-8/PDMS, Sun et al. [45] used molecular dynamics (MD) simulation, by constructing simulation models of three MMMs with increasing loadings. The results obtained were: (i) Strong attractive interaction between the ZIF-8 particles and the PDMS matrix, (ii) ZIF-8 loading rendered the PDMS chains less mobile, (iii) The experimentally found thermal properties corroborated those obtained through modelling. These modelling studies helped to understand the properties related to structure and diffusion across the MMM in-depth as these are difficult to understand through experiments due to the atomistic scale morphology of the membrane matrix along with the nanosecond timescale of diffusion. 

A lot of previous modelling studies do not take into account how the adsorption equilibrium at the interface between the polymer and the MOF particle affects the membrane permeability, as well as the effect of the presence of defects on the same. Singh et al. [46] have developed methods for automated construction of detailed and large-scale 3-D MMM models to show the importance of modelling in understanding the transport behaviour in MMMs. These were then solved by finite-element methods using the COMSOL Multiphysics package, while also providing extensive data plots and an accurate empirical correlation to accurately obtain reliable predictions for MMMs. The models explicitly account for the differences in molecular diffusivity between the matrix and the filler as well as the effects of interfacial equilibrium between the two phases. The results obtained have shown that the MMM performance is not affected by the particle size and, therefore, the dependency found on particle size in experiments could be attributed to some other indirect effects like enhanced interfacial interactions. The performance of permeability-based models was tested using the available CO_2_ solubility and diffusivity data for filler and polymer materials

Hydrocarbon permselective ZIF-8/PDMS membranes have been explored in the studies by Prajapati et al. [47]. Different membranes were prepared by varying the ZIF-8 loadings and the solvents. Ornstein-Zernike model, fit to their small-angle neutron scattering profiles, were used to observe the conformations of the polymer chains, which were found to vary with the ZIF-8 loadings. Larger scattering domains were observed for the membranes prepared in toluene than the membranes prepared in n-heptane of the similar ZIF-8 loading by the Debye-Anderson Brumberger model, which can be attributed to their different membrane nanostructures. This was done to understand the impact of the nanoparticle fillers on the structure-property relations of the MMMs, which are difficult to attain through experiments. 

### 2.6. Separation Process Involved

In studies conducted by Fan et al. [48], for the use of ZIF-8/PDMS membranes for solvent separation, three pathways were suggested for the passage of the solvent molecules through the selective layer: (i) through the polymer dense layer, (ii) through the inner channels of the filler nanoparticles due to their organophilicity and hydrophobicity and (iii) through the filler-polymer interface (gaps) [44]. In the pervaporation process, the permeation flux was greatly improved as the solvent/water mixture preferentially diffused through the inner channels of ZIF-8 nanoparticles and gaps as it offered lower resistance than the dense and nonporous PDMS layer. As a result, the permeation flux increased with the ZIF-8 loading, while the separation factor decreased as the greater number of gaps produced in the selective layer increased, increasing the probability of the diffusion of solvent molecules into the channels. The adsorption selectivity and framework flexibility of the ZIF-8 nanoparticles also helped improve PV performance. 

To improve the separation, new materials have also been investigated that allow for better separation routes. For instance, Zak et al. [49] have studied polymers with intrinsic microporosity (PIMs). The polymer structure within the membrane allows for greater internal surfaces and free volumes, thereby, allowing for better separation. Another way to improve the separation of solvents from ABE broth is by the method of combining the pervaporation with in situ fermentation [50].

Various ZIF-PDMS combinations applied for the solvent dehydration and solvent recovery are presented in Table 1. MMMs made from PDMS in combination with ZIF-7 for acetone/water [5], ZIF-90 for ethanol/water [10], ZIF-71 for butanol/water [12], ZIF-8 carbonized for butanol/water [15], MSS-ZIF71/ZIF-8 for ethanol/water [22], ZIF-8 for isopropanol/water [23] and ZIF-8 for butanol/water [31,33,34,36] and so on in the dehydration of solvents are presented in Table 1. Among them, ZIF-8/PDFMS showed comparatively better flux and separation factor [33,34] when compared to other ZIF materials.

## 3. Factors Affecting Pervaporation Performance of ZIF-PDMS Membranes

The factors affecting MMM performance were found to be ZIF loading [32,48], operating temperature [5,58], feed concentration [5], hours of operation [51,58], synthesis time [51] and filler particle size [41].

### 3.1. ZIF Loading

**Figure 9:**
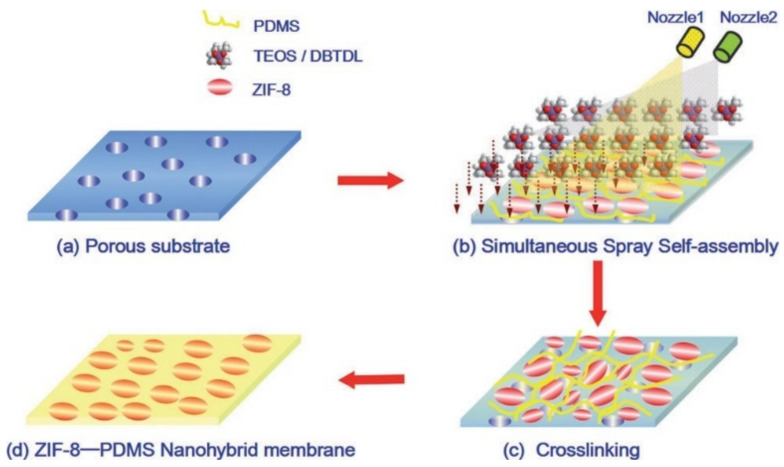
Simultaneous self-spray assembly method [48]—(Reprinted from Angewandte Chemie (International ed. in English), Fan, H.; Shi, Q.; Yan, H.; Ji, S.; Dong, J.; Zhang, G. Simultaneous Spray Self-Assembly of Highly Loaded ZIF-8-PDMS Nanohybrid Membranes Exhibiting Exceptionally High Biobutanol-Permselective Pervaporation., pp. 5578–5582, Copyright (2021), with permission from Elsevier).

It was shown that irrespective of the ZIF-8 loading, the ZIF-8 particles were homogeneously dispersed in the PDMS for the membrane prepared by simultaneous self-spray assembly technique, as shown in Figure 9. This was a result of the fact that there was no nanoparticle agglomeration due to the self-stirring in the pressure barrel which made the ZIF-PDMS suspension stable. On the PS support surface, the PDMS chains allowed the ZIF nanoparticles to remain separated and the spraying of the TEOS-DBTDL solution led to a cross-linking reaction. However, beyond 60 wt% loading, there was excess nanoparticle agglomeration and the separation factor was found to be reduced [48].

Jin et al. [32] observed a decline in PV performance of ZIF-8@PDMS membranes with 8 wt% loading, prepared for phenol separation via drying free process. At high loading, the probability of interaction and collision among the particles increased, severe particle aggregation occurred and large particles were formed, destroying the integrity of the hybrid membrane [37,44]. This increased the likelihood of formation of defects such as voids amongst the particles, as well as between the particles and the membrane, as investigated by Li et al. [36], on the PV performance of the O-ZIF-8@PDA/PDMS membranes for butanol pervaporation. These defects allowed greater mass transfer diffusion for the smaller water molecules, resulting in increased water permeability and decreased butanol permeability and selectivity. The results showed an increase in the total flux with the particle loading, while the separation factor and selectivity first increased, reached a maximum at 1 wt% loading and then began to decrease [44]. Similar behaviour was observed for ZIF-91 loaded PDMS as well, where both the flux and separation factor were increased on increasing the % loading (displaying an anti-trade off phenomenon) up to 20%, while both were found to decrease beyond 20% loading. The increase was expected to be due to a preferred pathway created by the ZIF-91 particles, whereas the later decline was due to interfacial defects and reduction of membrane-free volume [10]. 

The hydrophobic property of the membrane is also improved with increased particle loading [36]. As a result, a considerable increase in selectivity was achieved for PDMS/ZIF-8 membranes when used in butanol PV experiments up to 2 wt% loading, beyond which the selectivity was decreased, mainly due to incompatibility between the polymer and the ZIF particles at high loading concentrations. Both the water and butanol permeabilities were also decreased [55]. Similar hydrophobic behaviour was observed for the PDMS/DLA-ZIF-90 MMM. With an increase in particle loading, the fractional free volume increased, increasing the permeation flux. Up to 2.5 wt% loading, the sorption selectivity and polymer-filler compatibility were enhanced, increasing the separation factor, while it decreased beyond 2.5 wt% loading due to particle agglomeration and defects [21].

The ethanol and water permeabilities were increased with the ZIF-8 loading as the MMM was endowed with more transfer pathways for penetrants, improved hydrophobicity and ethanol affinity, also resulting in increased membrane selectivity [42]. An increase of flux observed by Li et al. [58] in the MAF-6/PDMS membranes was attributed to the same reasons of increased transfer channels. However, the separation factor was first found to increase then decrease. 

### 3.2. Operating Temperature

Few authors have studied the influence of pervaporation parameters such as temperature on the flux and separation performance of the membranes. Ying et al. [5] reported that both the total flux and the separation factor increased as the temperature was increased, as the transfer resistance decreased [58], promoting the passage of both acetone and water through the membrane. Thus, the PV performance enhanced with the increase in temperature. However, using high temperatures implies greater energy consumption, which is not economical. Thus, an optimization of temperature is required and is generally set slightly above the acetone boiling point. Similar results were observed by Fan et al. [31], where it was reported that the greater flexibility of polymer chains at high temperature made available larger free volumes of the PDMS crosslinking layer as well as in the PDMS/ZIF interface. An increase in separation factor was attributed to the increase in activation energy of the permeation component. Thus, the effect of temperature demonstrated the anti-trade off-trend in MMMs [10,33]. Similar results were obtained in other studies too [21,37,40,41,57]. On the other hand, the permeability was found to decrease, mainly due to the reduction in the solubility of the permeant components on the membrane surface, in the case of ethanol–water mixtures [21,44] as well as butanol–water mixtures [55].

It is to be noted here that the mobility of molecules was improved with increasing temperatures. Additionally, the vapour pressure difference on the two sides of the membrane increased, enhancing the driving force for PV. In certain cases, the flux increased with temperature, while the separation factor first increased and then declined [36,51,58].

### 3.3. Feed Concentration

Feed concentration is an important factor influencing the PV performance of MMMs. A “trade-off” relationship was found between the total flux and separation factor as the feed concentration was increased [5]. Few of the studies observed the effect of feed concentration as discussed below.

As the butanol content in the feed was increased, significant elevation in the total flux was observed resulting from an improvement in butanol flux, while having little effect on the butanol separation factor [51]. In another study, the separation factor was, however, found to decrease. As reported, the increase in butanol flux was due to an enhanced driving force because of its increased sorption in the membrane. This also increased the free volume and polymer chain flexibility, allowing water permeation through the membrane as well and this, along with the coupling effect originating from hydrogen bonding between water and n-butanol molecules, led to an increase in water flux too [31]. Similarly, for the pervaporation of ethanol, the permeate flux and selectivity were increased while the separation factor was decreased as the feed ethanol concentration increased. The reason, again, was increased sorption in the membrane [37]. This also resulted in membrane swelling, thereby, enhancing the flexibility of the polymer chain, decreasing the free volume cavities, lengthening the permeation pathways and, thus, limiting the molecular diffusion throughout the membrane [42]. Moreover, the dynamic radius of water is smaller than that for ethanol, so water could diffuse faster and offset the adsorption selectivity of ethanol [44,58].

The membrane prepared by Rao et al. [57] for separation of volatile aromatic compounds (VACs) from natural blackberry juice was investigated for the influence of concentration of linalool, benzaldehyde and ethyl acetate in the feed. As their concentrations in the feed increased, the flux for all three components increased. It was also attributed to increased sorption in the membrane, enhancing the driving force for the permeation. The separation factor for linalool and benzaldehyde decreased, following the “trade-off” trend. Contrastingly, the separation factor for ethyl acetate was found to increase, mainly assumed to be due to the smaller size and lower boiling point of ethyl acetate. However, with a further increase in concentration, ethyl acetate also followed the same downward trend. 

### 3.4. Hours of Operation

The ZIF/PDMS MMMs have displayed good long-term stability as shown in a few studies. It is one of the critical factors for the industrial application of membranes.

In a 240 h continuous operation period, the structural stability was conserved as the total flux and butanol concentration in permeate changed only slightly [51]. The O-ZIF-8@PDA/PDMS membrane exhibited a more or less constant total flux and separation factor for 240 h of PV performance [36]. ZNHT-PDMs membranes showed a 160 h cyclic stability, mainly due to enhanced interfacial compatibility, mechanical and thermal stabilities [40]. During a cyclic test of 130-h, the MMM showed long-term stability as well as reusability [42]. For the ZIF-8/PDMS membrane prepared by Li et al. [43], the total flux and separation factor were changed only slightly as tested during the 120 h continuous operation. Similarly, the performance of MAF-6/PDMS MMM was highly stable with a separation factor of more than 10 in a continuous pervaporation experiment of 120 h [58]. The 120 h cyclic stability of the membrane prepared by Zhu et al. [44] was attributed to the strong covalent bonding between the PDMS and AZIF-8, as improved by the cross-linking agent GOPTS. 

### 3.5. Synthesis Time

The increase in synthesis time from 3 min to 10 min rendered the membrane more hydrophobic with a high permeation flux. On further increase from 15 min to 30 min, both the permeation flux and separation factor were observed to decline, attributing to the greater thickness of the active layer and lower adsorption selectivity between water and ethanol as shown by Mao et al. [42]. To study the effect of membrane synthesis time on pervaporation performance, another attempt was made by varying the synthesis time from 0.5 to 4 h and it was observed that there was no effect on ZIF-71 particle size beyond 2 h and, thus, no significant effect on the performance of the membrane [52].

Synthesis time was also found to affect the particle morphology, in return affecting the membrane performance. As the synthesis time was increased to four hours, Naik et al. [22] found the MSS-ZIF-71 core-shell particles to be more homogeneous, while prolonging the time to 24 h resulted in the cracking of the shell layer of ZIF-71. 

### 3.6. ZIF Particle Size

Particle size also plays a vital role. It is generally challenging to predict how particle size exactly affects the MMM separation performance. Sizes of various ZIFs have been attempted to be controlled by altering different parameters (reactant ratio, sources of zinc, synthesis temperature and solvent, and additives) using several synthesis techniques [52].

Yin et al. [52] investigated the effect of particle size on membrane performance. The particle size is greatly affected by temperature and was found to increase with temperature, mainly due to reduced nucleation rates. The particle size distribution was also broader at higher temperatures. Reactant ratios did not have any significant effect on the particle size. By varying the synthesis time of the ZIF particles, it was found that nucleation and growth both occurred simultaneously in the first 1 h so a broad range of particles were obtained. Only growth and no nucleation occurred in the second hour, due to depletion of supersaturation, resulting in the formation of monodisperse particles. Beyond that, there was no effect on particle size. Larger particle size engenders less tortuous pathways in the membrane, thereby, providing less transport resistance as compared to smaller particles, which typically tend to agglomerate, introducing defects and decreasing membrane selectivity performance. Thus, with a larger particle size, there occurs a higher alcohol/water selectivity as well as higher alcohol and water permeabilities. 

The packaging density and particle size influences the geometry at the interface between the polymer matrix and the filler particles. The particle size was varied by changing the concentration of the precursor, Zn(NO_3_)_2_. With the increase in precursor concentration, the particle size reduced, whereas the number of particles increased, due to an enhanced nucleation rate. This was seen to have the following effects on the membrane performance: (i) continuous increase in separation factor and (ii) increase in permeation flux, reaching a maximum value and then a decrease [42].

### 3.7. Membrane Thickness

Membrane thickness is also an important factor affecting the separation performance of MMMs. Membrane thickness can vary due to the particle loading, nature of filler particles, particle size and other similar factors [59]. For instance, Li et al. [60] observed that the membrane thickness increases upon increasing the particle loading, thereby, affecting pervaporation performance. 

Zhu et al. [41] reported that a thinner selective layer resulted in a higher total flux, but a lower separation factor due to some inherent defects. It was observed that as the membrane thickness was decreased, the total flux increased gradually, while the separation factor increased and became steady at a particular value thereafter. A thicker membrane did not have a homogeneous morphology. Also, the real separation happened below the actual selective layer, and if the selective layer was thicker, the swelling effect of the dense layer on the membrane performance was reduced. However, with a greater reduction in membrane thickness, the resistance and mechanical strength of the membrane also deteriorated with the occurrence of greater defects, making the membrane unsuitable for practical applications [32].

## 4. ZIF-Polymer MMMs Involving Other Polymers

Membranes made of polymers other than PDMS have also shown to deliver good PV performance as shown in Table 2, covering the blending conditions, membrane characteristics as well as the separation performance. In light of the current environment conservation regulations, Castro-Munoz et al. [6] has presented a study on the use of biopolymers in the making of MMMs, replacing the synthetic ones. These polymers are generally obtained from sources like animals (e.g., poly(butylene succinate), poly(lactic acid), poly(hydroxyalcanoates)), vegetables (e.g., starch, cellulose-based polymers, alginate, polyisoprene), bacterial fermentation products (e.g., collagen, chitin, chitosan), and other specific production processes (e.g., sericin which is a by-product of the silk processing process). These polymers possess a high affinity towards polar compounds like water, can form films easily and can potentially be subject to chemical modification due to the large number of functional groups present. The only drawback is their low mechanical strength, which has nonetheless, been attempted to be improved by coating onto porous supports and crosslinking with other materials (i.e., glutaraldehyde and sulphuric acid), and physically merging with inorganic nanomaterials. Another biopolymer, that has been successfully employed for the separation of valuable metals in battery applications, in association with ZIF-8, with exceptional performance is K-Carrageenan [61]. With further investigation, such MMMs with sustainable materials can be used for solvent recovery and dehydration purposes too.

Various other combinations of polymer-ZIF membranes have also been inspected and are presented in Table 2. MMMs made from Poly(vinyl alcohol) in combination with ZIF-8 [62,63], those made using chitosan with ZIF [64,65] for alcohol/water separation, ZIF-8 with PEBA for phenol separation [66] and ZGO/PEBA membranes for butanol/water mixtures [67,68,69,70,71,72], and so on are presented in Table 2.Membranes that have been used for applications besides solvent dehydration and recovery have also been presented, like membranes used for removal of dyes from aqueous solutions [73,74,75,76,77,78,79], to show the good separation performance of these membranes and their potential use for solvent separation.

## 5. Transport Mechanism

The transport parameters are permeability (P), diffusivity (D) and solubility (S) where P = D×S = D×K. The solubility parameter provides the thermodynamic aspect at equilibrium conditions, with respect to the amount of the diffusing solvent adsorbed by the membrane, while diffusivity parameter provides the kinetic aspect with respect to the rate of transport of the diffusing solvent. D represents the diffusion coefficient while K represents the sorption coefficient [4].

The membrane performance is given by flux and separation factor. The flux (J) is given by J = m/At where m is the mass of the diffusing component, A is the transport area of the membrane and t is the collection time. The separation factor β is given by β = ((C_1_/C_2_)_permeate_)/(C_1_/C_2_)_feed_), the ratios of the two components in the permeate and the feed streams. The permeability can be defined as P = J*l/(p _A,G_ ^F^ − p _A,G_ ^P^), where the denominator terms are the partial vapour pressure terms and l is the thickness of the selective layer, whereas selectivity α is the efficiency of separation of the two components and is defined as the ratio of the permeabilities of the two components being separated [4,16,80].

### Maxwell Model

For a polymer-filler composite, Maxwell assumed a homogeneous sphere having a volume same as that of the composite of an infinitely diluted cluster of particles embedded in an infinite matrix with no particle interactions. The electrical conductivity of this composite was defined to be the same as that of the homogeneous sphere, as given by the equation β_fc_ = (α_fc_ -1)/(α_fc_ + 2) where α_fc_ = P_f_/P_c_ [81]. Attempts have been made to extend this model to spheroids [82] as well as to concentrated composites [83].

## 6. Conclusions

Mixed matrix membranes are the novel approach towards solvent separation and dehydration through the process of pervaporation. PDMS membranes have always been the go-to membranes for this, with their performance improved by incorporating nano-fillers like ZIF particles into the membrane. Several studies have been conducted to overcome the issues faced by the MMMs, to obtain superhydrophobic membranes, to modify the ZIF nanoparticles for better performance and to synthesize the membranes using novel techniques. Several factors affect the pervaporation performance of the synthesized membranes including the ZIF nanoparticle size, the operating temperature, the concentration of the ZIF in the membranes, the membrane thickness, the time of operation, the concentration of the feed solution as well as the synthesis time for the membrane. Studies have also been conducted on the use of polymers besides PDMS that have been used as a matrix for ZIF particles to form membranes. A detailed review has been provided on the kind of membrane prepared, the blending conditions, the membrane characteristics, and the pervaporation performance, along with some recommendations for future research to be carried out in this field.

The two most important solvents are ethanol and butanol that need to be separated from fermentation broths for aqueous solutions. The best method for ethanol pervaporation was that with ZIF-91/PDMS membrane at 55 °C, while ZIF-71/PDMS also showed good performance when doped with mesoporous silica spheres, which was fabricated using hexane as the solvent with ultrasonication at 60 °C. For butanol, ZIF-8/PDMS membranes with SAM modification as well as O-ZIF-8@PDA/PDMS membranes fabricated using the drying free process was found to be the best. 

## 7. Future Outlook

The review presents certain future aspects to be researched upon:

(1) Ways to tailor the morphology and functional groups of the MOFs to improve MOF-polymer interactions. The morphology of the fillers can be changed from spherical to lamellar or fibrous-shaped, for better performance. Functional groups can be changed to make the MOFs dynamic/responsive to external stimuli like temperature and pressure [18]. Low molecular weight coupling agents can also be used for better polymer-MOF interactions [19]. Methods of priming and thermal annealing can also be used [84].
(2) Process of tuning the micro-structure of the filler particles, keeping in mind the crystallinity and fractional free volume. One of the methods to do this is to make the pore orientation parallel to the direction of gas diffusion. However, it is difficult to achieve and a method is required for the growth of the particles along with the desired pore orientation [18].
(3) Methods to select the best MOF-polymer combination and studying the effect of the MOF on the corresponding polymer. For instance, choosing a polymer that allows for a better porous structure when combined with the inorganic fillers can help improve separation [18].
(4) Finding more stable and chemically resistant membrane materials. Generally, MOFs are vulnerable to acidic conditions. For instance, nano-sized fillers resistant to aggregation and harsh chemical conditions should be looked into [18].
(5) Performing modelling studies to predict MMM performance. There are only a few studies that take into account the presence of interface defects and their effect on membrane performance. Such studies might be difficult to perform experimentally, and, thus, need molecular simulations as the role of the interface is important in the diffusion through the membrane [85]. Modelling studies can also help identify compatible MOF-polymer pairs as well as ways to improve the compatibility for the pairs that are not [86].
(6) Optimization of the membrane structure to reduce mass-transfer resistance [26]. This can be done through modelling studies.
(7) Exploring the concept of self-healing for membranes used in solvent separation and dehydration. For instance, a self-healing membrane has been developed for the separation of oil from wastewater using a ZIF-PDMS membrane modified with multi-walled carbon nanotube film. Such a membrane was able to handle harsh environmental conditions and perform self-healing upon external damage [87].

## Figures and Tables

**Table 1:** ZIF-PDMS mixed matrix membranes’ blending conditions, membrane characteristics and separation performance.

Sl.no.	ZIF Type	PDMS ZIF/PDMS/Solvent	MW	Blending Conditions	Size of ZIF Particles/MMM Thickness,% of ZIF Loading	Solvent Systems (S.F.-Separation Factor)	References
1.	ZIF-7 Superhydrophobic	Heptane	10,000 g/mol PDMS	ZIF-7 in n-heptane with stirring for 6 h. PDMS added and stirred for 4 h. Tetraethyl orthosilicate (TEOS) and dibutyltin dilaurate (DBTDL) added. PDMS:TEOS:DBTDL = 10:1:0.1. Polyvinylidene difluoride (PVDF) film used as the support. Membrane dried in a vacuum oven for 8 h.	80 nm sized particles 0–40 wt% loading	Acetone/water at 333 K (40 wt% loading) Flux-1542.6 g/m^2^ h (25 wt% loading) Flux-1236.8 g/m^2^ h SF-39.1	[5]
2.	ZIF-90 ZIF-91 ZIF-92	Tetrahydrofuran (THF)	3000 mPa.s viscous PDMS	ZIF added into PDMS/THF solution and stirred vigorously for 24 h. Curing agent (TEOS) and catalyst DBTDL added under magnetic stirring at room temperature for 2 h. Solution degassed and applied on the PVDF substrate with a casting knife. Dried overnight at 60 °C on vacuum condition, followed by annealing of fabricated MMMs at 80 °C for 2 h.	Average diameters: 132 nm (ZIF-90), 275 nm (ZIF-91) and 1208 nm (ZIF-92) 20 wt% loading	5 wt% ethanol-water solution at 55 °C Best:ZIF-91/PDMS Flux-846 g/m^2^ h SF-15.8	[10]
3.	ZIF-71 Hydrophobic	Heptane	110,000 g/mol PDMS	PDMS-heptane solution. ZIF-71-heptane solution. Two solutions mixed. TEOS added. Titanium 2-ethylhexoxide and di-n-butyldiacetoxytin tech-95 added. Film poured on Teflon flat dish and dried.	646.2 ± 6.3 nm sized particles 100–300 µm thick membrane 40 wt% loading	1-butanol/water SF-69.9 ± 1.8 Ethanol/water SF-9.2 ± 0.7	[12]
4.	ZIF-8 Carbonized-hydrophobic	Hexane ZNC/PDMS	5000 mPa s Viscous PDMS	PDMS+ZNC particles+TEOS mixed in n-hexane. DBTDL added and the mixture stirred and degassed. Casting solution cast on a piece of polyvinylidene fluoride (PVDF) membrane.	9.53 μm to 11.96 μm thick membrane 10 wt% loading	ABE fermentation brothFlux-1870 g/m^2^ h SF- 16.8 (acetone) 4.5 (ethanol) 20.7 (n-butanol)	[13]
5.	ZIF-8 Carbonized	ZNC/PDMS Hexane	5000 mPa s Viscous PDMS	PDMS+ZNC particles+ TEOS mixed in n-hexane and stirred for 2 h. PDMS and TEOS was 18:1. DBTDL added and the mixture stirred for 5 min and degassed. Casting solution cast on a piece of polyvinylidene fluoride (PVDF) membrane.	3 wt% loading	1.5 wt% butanol/water solution at 55 °C Flux-1249.5 g/m^2^ h SF-53.1	[15]
6.	ZIF-90 dodecylamine-modified ((DLA-ZIF-90) Hydrophobic	Hexane	NA	PVDF membrane, fabricated by the non-solvent induced phase inversion process, was used as the support for the preparation of PDMS MMMs. Particles added to PDMS/hexane solution with 12 h vigorous stirring and 2 h sonication. 10 wt% curing agent added at 60 °C with 2 h stirring. Overnight degassing, casting on PVDF support and drying at 60 °C under vacuum overnight. Annealed at 100 °C for another 6 h	500 nm sized particles Thickness: 107 µm (1 wt% loading), 100 µm (2.5 wt% loading), 125 µm (5 wt% loading) 1, 2.5 and 5 wt% loadings Optimum- 2.5 wt% loading	Ethanol recovery at 60 °C 5/95 wt% ethanol/water mixture 2.5 wt% loading: Flux-99.5 g/m^2^ h SF-15.1	[21]
7.	MSS-ZIF-71 and MSS-ZIF-8 (mesoporous silica spheres)	Hexane	NA	Two components of the PDMS (RTV-615 A and B, prepolymer and cross-linker, respectively) dissolved separately in hexane. The MSS–ZIF nanoparticles dispersed ultrasonically in hexane for 1 h. Prepolymer, the cross-linker and the filler mixed and stirred at 60 °C for 4 h. Solution poured into a glass petri dish, kept in an oven at 110 °C for at least 1 h.	2–3 µm sized particles 10 µm thick membrane 10, 15, 20 wt% loading	6 wt% Ethanol/water (with 20 wt% loading) MSS-ZIF-71: Flux-1000 g/m^2^ h SF-13 MSS-ZIF-8: Flux-720 g/m^2^ h SF-15	[22]
8.	ZIF-8	Hexane PDMS–ZIF/PI–ICA	NA	Polyimide substrate prepared and washed with methanol and soaked with 10 wt% ethylenediamine methanol solution at room temperature for 10 min followed by immersion in 1 wt% ICA methanol solution at 60 °C for 5 min followed by horizontal immersion in Zn(CH_3_COO)_2_·2H_2_O solution and 1 min sonication. Hmim and ammonia hydroxide solutions added. Ultrasonication for 5 min. Overnight crystallisation and washing with water. Membrane soaked in secondary growth solution (mixture of Zn(NO_3_)_2_·6H_2_O and Hmim solution) for 6 hr at room temp, washed and soaked in PDMS solution (5 wt% in hexane) for 3 min.	40–50 nm sized particles 190.4 nm thick skin layer	Isopropanol dehydration 85/15 wt% IPA/water mixture at 40 °C	[23]
9.	ZIF-8	Heptane 10 wt% PDMS	20,000 Pa s Viscous PDMS	Polysulfone (PS) ultrafiltration sheet supports. Non-dried ZIF-8/ethanol suspensions dispersed dropwise in dilute PDMS/n-heptane solution (labeled as suspension-dispersed ZIF-8/PDMS). TEOS (1 wt%) and DBTDL (0.05 wt%) added and stirred for 0.5 h. A concentrated PDMS pre-cross-linked solution (10 wt%) prepared. First, a homogeneous ZIF8/PDMS membrane formed by repeatedly and horizontally dipping the pre-treated PS supporting membrane in the ZIF-8/PDMS solution (1 wt%) (1 min immersion per layer, 30 s intervals), followed by fixing perpendicularly onto a substrate with a rotating motor with continuous baking by a burner to remove the residual solution on the support surface. Membrane dipped once in the concentrated PDMS. Membranes allowed to stand for 1 day in air at room temperature, then placed in a convection oven at 80 °C for 8 h.	1 µm sized particles 1.8 µm thick membrane	5.0 wt% n-butanol–water solution at 80 °C Flux-2500.8 g/m^2^ hSF-52.81	[31]
10.	ZIF-8	ZIF-8@PDMS/PVDF nanofibrous composite membrane Hexane 10 wt% PDMS	18,000–22,000 cSt viscous PDMS	Drying free process: PDMS/hexane solution prepared. ZIF/hexane solution prepared. PDMS solution added dropwise followed by vigorous stirring for 1 h. Cross-linker TEOS (5 wt%) and catalyst DBTDL (1 wt%) added. Solution poured into an aluminium petri dish. Complete solvent evaporation at room temperature followed by heat cured in an oven at 80 °C for 24 h. Electrospun PVDF nanofibrous membrane used as support.	180 nm sized particles ZIF-8@PDMS: 400 ± 30 µm thick membranes ZIF-8@PDMS/PVDF: 13.2 ± 1.1 µm thickness 0, 1, 4, 8 wt% loading (4 wt% optimum)	Phenol separation in the aqueous-aqueous membrane extraction process ZIF-8@PDMS: k_0_ of 2.61 ± 0.05 × 10^−7^ m/s ZIF-8@PDMS/PVDF: k_0_ of 35.7 ± 1.1 × 10^−7^ m/s	[32]
11.	ZIF-8	No solvent	300 mPa·s, viscous and 14,000 g/mol PDMS	Polymethylhydrosiloxane (PMHS) (cross-linker) and ZIF dissolved in ethanol, platinum catalyst added dropwise. PDMS and prepared suspension repeatedly and alternately sprayed onto a rotating Polysulfone membrane surface using two horizontal spray nozzles at 80 °C	90 nm sized particles 6 µm thick selective layer 0–15 wt% loading	1–5 wt% aqueous butanol solution at 30–70 °C Flux-2334.6 g/m^2^ h SF-64.5	[33]
12.	ZIF-8	ZIF/PDMS ZIF/PDMS/SF	NA	Dip-coating and SAM modification. Membrane stable underwater even after 7 days of immersion.	80–90 nm sized particles	n-butanol/water (with SAM modification) Flux-1339 g/m^2^ h SF-84.8	[34]
13.	ZIF-L	Hexane, Water	NA	3-D printed PA membrane. Membrane coated with ZIF-L solution and dried for 12 h at 60 C. Resulting membrane coated with Hmim and zinc nitrate solutions to obtain multiscale layers. PDMS solution in n-hexane prepared. Above membrane coated with the solution by immersion for 10 min. Resulting membrane allowed to solidify at 70 °C for 30 min in the oven.	2.7–7.2 µm sized particles	Oil/water Flux- 24,000 L/m^2^.h	[35]
14.	Silane modified ZIF-8 P-ZIF-8@PDA and O-ZIF-8@PDA Hydrophobic	Hexane P-ZIF-8@PDA/PDMS and O-ZIF-8@PDA/PDMS	5000 mPa s viscous PDMS	Particles, PDMS and TEOS added in n-hexane and stirred for 2 h. PDMS:TEOS = 18:1. DBTDL added and stirred for 5 min. Casting solution coated on a PVDF membrane using an automatic film applicator followed by curing at room temperature for 24 h.	11.5 µm thick membranes 1 wt% loading	1.5 wt% butanol solution at 55 °C O-ZIF-8 @PDA/PDMS: Flux-480.6 g/m^2^ h SF-56 P-ZIF-8 @PDA/PDMS: Flux-480.6 g/m^2^ h SF-47	[36]
15.	MCM-41@ZIF-8	Heptane	PDMS kinetic viscosity of 3000 mPa s	Casting solution poured into the surface of PS membrane. The composite membrane was prepared by a doctor blading method. After standing at room temperature for 12 h, the composite membrane was transferred to a vacuum oven at 90 °C for 12 h to fully cross-link	20–30 nm sized particles 3 µm thick selective layer 5 wt% loading	5.0 wt% ethanol/water at 70 °C Flux-2204 g/m^2^ h SF-10.4 3 wt% n-butanol/water at 60 °C Flux-2052 g/m^2^ h SF-45	[37]
16.	ZIF-L nanosheets	Heptane	5000 mPa s viscous PDMS	ZIF-L dispersed in n-heptane using probe sonicator. 10 wt% PDMS added followed by stirring. Cross-linking and binding agent (3-aminopropyl) triethoxysilane (APTES) and catalyst DBTDL added such that 1:0.1:0.05:4 (PDMS: APTES: DBTDL: n-heptane). Solution cast onto the PVDF support. Evaporation of the residual solvent and crosslinked at 120 °C for 3 h.	ZIF-L sheets: dimensions of about 5.6 μm × 2.2 μm and a thickness of about 136 nm. Active layer thickness for each membrane was averaged to be 8.8 μm (10 wt%), 14 μm (20 wt%), 17.3 μm (30 wt%), 23.2 μm (40 wt%), and 38.7 μm (50 wt%) 10,20,30,40,50 wt% loadings	5 wt% Aqueous alcohol solutions (ethanol, n-propanol or n-butanol) at 40 °C 1.0 wt% n-butanol aqueous solution at 40 °C Flux-402 g/m^2^ h SF-57.6	[38]
17.	ZIF-71	Heptane	110,000 g/mol PDMS	PDMS/heptane solution added dropwise to ZIF-71-heptane suspension with sonication. After some amount of solvent removal, TEOS added and stirred. TEOS and DBTDL added. Mixture poured into a Teflon flat dish in a humidity-controlled box (75% RH). After 21 h, the films removed and dried in two stages in a vacuum oven; first at 100 °C for 20 h and second in at 120 °C for 11 h.	506 nm sized particles 25 wt% loading	2 wt% ethanol/water or 1-butanol/water solution at 60 °C	[39]
18.	ZIF-8 ZIF-8-capped halloysite nanotubes (ZHNT)	Heptane	50 Pa.s viscous PDMS	ZHNTs dispersed ultrasonically in n-heptane for 1 h. ‘‘Primed’’ by introducing a certain content of PDMS and stirred for 6 h. Remaining PDMS added and stirred for 6 h. TEOS and DBTDL added (PDMS: TEOS: catalyst: n-heptane—1:0.1:0.05:9). Solution cast onto the PVDF substrate. Membranes placed for 15 min at 120 °C for 3 h.	ZHNT: outer diameters of 30–60 nm, inner diameter about 25 nm, length of 400–750 nm The average thickness of separation layer: 10.7 μm (5 wt%), 11.1 μm (10 wt%), 12.2 μm (15 wt%), and 14.8 μm (20 wt%)	1 wt% n-butanol aqueous solution at 40 °C Flux-683 g/m^2^ h SF-61.3	[40]
19.	ZIF-8@GO	THF	1000 cst viscous PDMS	Doping particles dispersed in THF by sonication and stirring for 12 h. PDMS and TEOS added, followed by sonication for 1 h and stirring for 7 h. Catalyst DBTDL added and stirred for 30 min. After degassing, the solution cast onto the PVDF support layer. Membrane cured at 30 °C for 12 h, and at 80 °C for 6 h.	Less than 50 nm sized particles 9.2 µm thickness 0.75 wt% loading	5 wt% ethanol aqueous solution at 40 °C Flux-443.8 g/m2 h SF-22.2	[41]
20.	ZIF-8	Methanol, ethanol, heptane	5000 mPa s viscous PDMS	Hmim/methanol/ethanol solution with vigorous stirring. The PDMS/n-heptane solution, cross-linker APTES added, followed by stirring and sonicating alternately for 0.5 h and the catalyst DBTDL was added. After degassing, the resultant organic phase was dip-coated on the PVDF support. Membrane placed in air atmosphere for 10 min and dried sequentially in a 120 °C oven for another 3 h followed by rinsing with methanol and drying.	1 µm thick active layer	5.0 wt% ethanol aqueous solution at 40 °C. Flux-1778 g/m^2^ h SF-12.1	[42]
21.	ZIF-8	Hexane	NA	Firstly, Solution A (PDMS/TEOS) and solution B (PDMS/DBTDL) were coated on the PVDF substrate to form the PDMS layer. Then the solution C (PDMS/Zn(NO_3_)_2_) and solution D (PDMS/Hmim) were coated on the surface of the PDMS layer in turn. Subsequently, the obtained MMMs were thermally cured at 80 °C for 2 h. The spin-casting procedure operated last for 30 s at 1500 rpm.	Average thickness 17 µm of the active layer 10, 20, 30 wt% loadings	1.5 wt% n-butanol aqueous solution at 55 °C 20 wt% loading: Flux-2046.3 g/m^2^ h SF-42.6	[43]
22.	AZIF-8 (amine-functionalized)	Hexane	Silanol-terminated PDMS (3000 cst, density of 0.98 g/cm^3^)	ZIF/n-hexane suspension transferred into PDMS/n-hexane solution, followed by vigorous stirring for 5 h, sonication for 1 h, and 2 h stir. Crosslinker GOPTS and catalyst DBTDL added, stirred vigorously for several minutes. After degassing, the mixed solution was cast onto the PVDF support layer, cured at 40 °C for 12 h, and heated to 60 °C for 6 h.	100 nm sized particles 6.8 µm membrane thickness 7 wt% loading	5 wt% aqueous ethanol solution at 40 °C Flux-585.6 g/m^2^ h SF-17.7	[44]
23.	ZIF-8	Heptane	NA	ZIF dispersed in n-heptane (ultrasonication with stirring for 2 h), then PDMS (10 wt%) added. Stirring for 1 h. (ZIF:PDMS = 10:60 wt%). Cross-linking agent TEOS (1 wt%)+ catalyst DBTDL (0.05 wt%) dissolved in n-heptane plus stirring at room temp for 1 h. Simultaneous spray self-assembly on sheet polysulfone substrate from two separate barrels with controlled spraying. Vacuum oven at 80 °C for 8 h.	Ultrathin nanohybrid selective layer 800 nm thick top selective layer 10–40% loading	1 wt% aqueous butanol solution at 80 °C (with 40%loading) Flux-4846.2 g/m^2^ h SF-81.6 45 wt% of n-butanol recovered	[48]
24.	ZIF-7	THF	kinetic viscosity, 20,000 mPa s of PDMS	PDMS in THF and stirring for 3 h. ZIF in THF sonicated for 30 min. Both solutions mixed and stirred for 1 h. TEOS and DBTDL added such that 5:1:0.4:20 (PDMS: TEOS: catalyst: THF). Solution poured on the PVDF ultrafiltration membrane and cast with a scraper. Dried overnight and treated at 80 °C for 4 h.	80 nm sized particles 20 µm thick top selective layer 20 wt% loading	1 wt% butanol aqueous solution at 60 °C Flux-1689 g/m^2^ h SF-66	[51]
25.	ZIF-71	Heptane	10,000 g/mol PDMS	Condensation cured membrane. PDMS-heptane solution prepared. ZIF-71-heptane solution prepared with vortex mixing. Both solutions mixed with sonication and vortex mixing. Resulting solution stirred to evaporate the heptane. TEOS and catalyst added. Solution poured on Teflon dish and allowed to dry.	Varying sizes: 152 ± 45 nm, 506 ± 28 nm, and 1030 ± 385 nm 140–390 µm thick membranes 25 wt% loading	2 wt% 1-butanol/water at 60 °C SF-63 2 wt% 1-ethanol/water at 60 °C SF-12.2	[52]
26.	ZIF-8	Heptane	NA	Hybrid hollow fibre membranes. ZIF-heptane solution and PDMS-heptane solution mixed. TEOS and DBTDL added. Mass ratios of ZIF-8 to PDMS set at 10–40%. Resulting solution coated on the inner surface of polyacrylonitrile (PAN) hollow fibres and heated at 60 °C to allow crosslinking.	100 nm sized particles 5 µm thick membrane 10–40 wt% loading	Isopropanol/water distillation	[53]
27.	ZIF-8	THF	50,000 mPa·s viscous PDMS	PDMS-THF solution prepared with stirring for 2 h. ZIF-THF solution prepared with sonication for 20 min in an ice bath followed by warming to room temperature. PDMS solution added to ZIF solution followed by sonication for 10 min. TEOS and catalyst added such that PDMS:TEOS:Catalyst = 100:4:1. Stirring for 2 h followed by casting on PVDF support membrane.	70 nm sized particles 25 µm thick membrane 0–20 wt% loading (optimum- 10 wt%)	20%/80% (vol/vol) Propane/nitrogen (For 10 wt% loading) SF-20.5	[54]
28.	ZIF-8	DMF	NA	ZIF added to PDMS solution and stirred for 2 h. Solution cast with a knife on a polyethersulfone (PES) ultrafiltration membrane and introduced into a vacuum oven at 80 °C for 20 h for a complete cross-linking reaction.	50–100 nm sized particles 73 µm thick membrane with 2 wt% loading 1–5 wt% loading (optimum: 2 wt%)	0.96 wt% n-butanol aqueous solution at 30 °C	[55]
29.	ZIF-67	Toluene-hexane	NA	Selective layer of PDMS dip-coated on the support. PDMS solution in toluene and hexane (80:20) (RTV 615 A and 615B in a 10:1 ratio) pre-crosslinked for 2 h at 60 °C. Coating solutions loaded with filler concentrations, stirred and sonicated for 15 min each (3 times). The support plate kept at an angle of 60° and PDMS solution poured on the support. This step was repeated at least three times allowing solvents to evaporate for 5 min in between. The cross-linking was completed in an oven at 110 °C for 24 h.	200 nm sized particles 5 wt%, 10 wt%, 15 wt% and 20 wt% loadings	6 wt% aqueous ethanol solution at a temperature range of 40–70 °C 20 wt% loading at 40 C: Flux-2780 g/m^2^ h SF-15.4	[56]
30.	ZIF-8	PDMS 20 wt% Tetrahydrofuran	50,000 mPa·s viscous PDMS	ZIF-8 mixed with THF and stirred for 30 min. PDMS added and stirred for 2 h. Ultrasound performed for 15 min. TEOS and a catalyst added at a mass ratio of 100:4:1. Stirred then allowed to cross-link for 2 h. Solution stirred then poured onto the PVDF membrane. Left at room temperature for 12 h, transferred to a vacuum oven at 130 °C for 2 h.	60 nm sized particles 3 µm thick membranes 6 wt% loading	Volatile aromatic compounds (VACs) from natural blackberry juice	[57]
31.	ZIF-6 (MAF-6)	Heptane	60,000 g/mol PDMS	MAF-6/n-heptane suspension stirred and sonicated alternatively for 1/2 h for three times each. Small amount of PDMS added and stirred for 2 h. Suspension mixed with cross-linked PDMS solution (PDMS, n-heptane, TEOS and DBTDL) and stirred for 3–4 h. Membranes cast on PVDF substrate, evaporated at room temperature for 24 h and dried in oven at 70 °C for 12 h.	150 nm sized particles 5 µm thick membranes 0, 5, 10, 15, 20, 25 wt% loadings	Ethanol/water mixtures at 40 °C 15 wt% loading: Flux-1200 g/m^2^ h SF-14.9	[58]

**Table 2:** ZIF-Polymer mixed matrix membranes’ blending conditions, membrane characteristics and separation performance.

Sl.no.	ZIF Type	Polymer/ MMM	Solvent	Blending Conditions	Size Size of ZIF Particles/MMM Thickness, % of ZIF Loading	Solvent Systems (S.F.-Separation Factor)	References
1.	ZIF-8 and ZIF-8-MCM-41 core-shell particles (MSS-Z8)	Polyimide Matrimid^®^ 5218	Chloroform	Fillers dispersed in the solvent in an ultrasonic bath for 20 min and stirred overnight. Polymer added and stirred magnetically at room temperature for 24 h, followed by sonication. Solution cast on a Petri dish and left covered overnight followed by 24 h in a vacuum oven at 180 °C.	0.17 ± 0.02 µm ZIF-8 4.3 ± 0.6 µm MSS-Z8 112 ± 10 µm ZIF-8 MMM125 µm MSS-Z8 MMM 12 wt% loading	10/90 wt% water/ethanol mixtures at 42 °C ZIF-8 MMM Flux-260 g/m^2^ h SF-300 MSS-Z8 MM Flux-200 g/m^2^ h SF-137	[7]
2.	ZIF-71 Hydrophobic	Polyether-block-amide (PEBA)	n-butanol	ZIF-71 particles dispersed in n-butanol, stirred and sonicated alternatively for 1/2 h for three times each. “Primed” by adding a small amount of PEBA stirred at 80 °C for 4 h. Remaining polymer added and stirred for 4 h. Solution kept at 60 °C overnight. Membranes cast on PVDF substrate. After 2 days, dried in an oven at 70 °C for 24 h.	1 µm particle size 10–20 µm thick membrane 20 wt% loading	Biobutanol recovery from acetone–butanol–ethanol (ABE) fermentation broth at 37 °C Flux-447.9 g/m^2^ h SF-18.4	[14]
3.	ZIF-8-NH_2_	Poly(vinyl alcohol) (PVA) PVA/ZIF-8-NH_2_	Water	PVA solution prepared at 90 °C by stirring and complete dissolution. ZIF-8 particle suspension in DI water prepared, shook for 1 h vigorously, and ultrasonicated for 1 h. Suspension added into PVA solution, stirred for 12 h vigorously ultrasonicated for 30 min. Overnight degassing. Solution cast on a polyethylene terephthalate (PET) plate and dried at room temperature under vacuum overnight. Peeled and dried at 50 °C for 12 h.	200 nm-sized particles 15 µm thick membranes 7.5 wt% loading	Ethanol dehydration (85/15 wt% ethanol/water mixture) at 50 °C Flux-185 g/m^2^ h SF-119 85/15 wt% isopropanol/water mixture at 40 °C Flux-112 g/m^2^ h SF-1200	[62]
4.	ZIF-8	Poly (vinyl alcohol)PVA/ZIF-8	Water	Drying free process and water phase solution	60 nm sized particles 20–50 µm thick membranes 0–39 wt% loading	Ethanol dehydration ethanol/water mixture (90:10 *w*/*w*) at 25 °C 39 wt% loading SF-4725	[63]
5.	ZIF-8	Chitosan CS/ZIF-8	Acetic acid	ZIF-8 heated at 120 °C under vacuum overnight, then dispersed in 2 wt% acetic acid aqueous solution. 10% of the desired amount of CS added to the ZIF-8/solvent suspension and stirred overnight. Remaining CS dissolved in the previously prepared solution to reach a CS concentration of 2 wt% and stirred for 24 h. The solution was degassed under vacuum (–0.04 MPa) at room temperature for 4 h and reacted with GA as a cross-linker. Defined volumes of the resulting solution cast on a glass plate and dried at room temperature for 48 h.	60 nm sized particles 40–60 µm thick membrane 2.5, 5, 7.5, 10 wt% loading	Isopropanol dehydration 85 wt% IPA aqueous solution at 30 °C 5 wt% loading Flux-410 g/m^2^ h SF-723	[64]
6.	ZIF-7	Chitosan ZIF-7/CS	Water, acetic acid	CS powders dissolved in DI with 2 wt% acetic acid. ZIF-7 particles (heated at 160 °C for 24 h) added, followed by vigorous stirring for 12 h and reaction with GA in an ice-water bath for 0.5 h. The solution was cast to form a thin film, dried in an oven at 45 °C overnight and then peeled.	1–2 µm sized particles 18 µm thick membrane 2.5, 4, 5, 6 and 7.5 wt% loadings (optimum- 5 wt%)	90 wt% ethanol aqueous solution at 25 °C 5 wt% loading: Flux-322 g/m^2^ h SF-2812	[65]
7.	ZIF-8	Polyether block amide (PEBA-2533)	N,N-dimethyl acetamide	ZIF-8 particles (dried at 100 °C for 1 h in a vacuum oven) mixed with N,N-dimethyl acetamide, followed by stirring and sonication alternatively for 0.5 h for three times. PEBA-2533 added and stirred at 70 °C for 1.5 h and sonicated at 70 °C. Solution cast onto a glass plate at 70 °C, followed heating at 70 °C in an oven for 1 day, followed by a vacuum oven at 50 °C for about 24 h.	100 nm sized particles 50 µm thick membrane	0.8 wt% Phenol aqueous solution at 70 °C 10 wt% loading: Flux-1310 g/m^2^ h SF-53	[66]
8.	ZIF-90	Polyvinylidene difluoride (PVDF)	DMF, acetone	PVDF dissolved in DMF. ZIF dissolved in acetone and ultrasonicated for 10 min. Both solutions mixed and ultrasonicated. Magnetic stirring on a hot plate for 50 °C. Solution cast on a glass substrate. Dried at 70 °C for 20 min.	100 nm sized particles 40–60 µm thick membranes 5, 10, 20, 30 wt% loading	NA	[67]
9.	ZIF-8	Polybenzimidazole (PBI) PBI/ZIF-8	1-Methyl-2-pyrrolidinone (NMP)	PBI dissolved in NMP by stirring for 48 h at 120 °C, followed by cooling down to room temperature and filtration. ZIF-8 dispersed in NMP. Stirred and sonicated. Added into a PBI/NMP solution followed by stirring. Solution poured into a casting ring on a silica wafer and dried in a vacuum oven at 75 °C for 12 h. Membrane peeled and dried in a vacuum oven at 200 °C for 12 h.	50 nm sized particles50 µm membrane thickness12.4, 27.4 33.7, 58.7 wt% loadings	Alcohol/water mixture of 85/15 wt/wt at 60 °C 33.7 wt%: IPAFlux-103 g/m^2^ h SF-1686 Ethanol Flux-81 g/m^2^ h SF-3417 n-butanol Flux-992 g/m^2^ h SF-10 58.7 wt%: IPA Flux-246 g/m^2^ h SF-310 n-butanol Flux-226 g/m^2^ h SF-698	[68]
10.	PEG-g-ZIF-8 Polyethylene glycol grafted ZIF-8	Poly(vinyl alcohol) PEG-g-ZIF-8/PVA	Water	Solution casting and solvent evaporation method with GA as cross-linking agent.	25 nm sized particles 40 µm thick membranes 5, 10, 15 wt% loading	88:12 wt/wt Isopropanol/water solution at 25 °C 15 wt% loading Flux-91 g/m^2^ h SF-7326	[69]
11.	ZIF-8	PVDF ZIF-8/gelatin/PVDF	Ethanol/water	ZIF-8 seeds/gelatin layer prepared by immersing ZHNs/gelatin/PVDF hollow fibre into Hmim ethanol/water solution at room temperature for 12 h followed by immersion into Zn(NO_3_)_2_·6H_2_O and Hmim solution at 30 °C for 6 h.	2 µm thick membranes	Rhodamine B dye/water Rejection-90.5% Permeance-137 L/m^2^ h bar	[70]
12.	GO@ZIF-67	Polyacrylonitrile (PAN)GO@ZIF-67/PAN	DMF	Casting method. GO@ZIF-67 added to DMF and sonicated for 5 min. PAN powder added to the mixture followed by stirring for 12 h. Solution poured into a glass petri dish and dried at 80 °C for 4, cooled to room temperature and immersed in DI water and dried.	NA	Methylene blue/water Photocatalytic adsorption	[71]
13.	ZIF-8 modified graphene oxide (ZGO)	Polyether block amide (PEBA) ZGO/PEBA	Methanol, n-butanol	ZGO in methanol and 2 h sonication and 4 h stirring. PEBA in n-butanol and 4 h mixing at 80 °C. ZGO laminates deposited on the surface of the ceramic substrates by vacuum-assisted assembly method in 3 min. Immersed into PEBA solution for 3 min. Stabilized with vacuum suction in air for 3 min. Dried for 5 h at different temperatures.	1 µm thick membrane	1% butanol from water at 75 °C Flux-606 g/m^2^ h SF-23.7 5% butanol from water at 55 °C Flux-1001 g/m^2^ h SF-29.3	[72]
14.	ZIF-90	Poly(vinyl alcohol) (PVA) ZIF-90/PVA	Water	Viscosity-driven in situ self-assembly method. PVA dissolved in DI water at 90 °C for 1 h with stirring. ICA solution added at 60 °C with stirring for 30 min. Zn(NO_3_)_2_·6H_2_O solution added and stirred for 2 min. Solution cast onto the culture dish at room temperature. Peeled off. Thermal treatment at 90, 110, 130, and 150 °C in a vacuum oven for 2 h.	350 nm sized particles 70–80 µm thick membrane	90 wt% ethanol aqueous solution at 30 °C Flux-268 g/m^2^ h SF-1379	[73]
15.	ZIF-71	PVDF ZIF-71/PVDF hollow fibre membrane	DMF	Dilute solution phase inversion process. PVDF powders and PEG-400 dissolved in DMF followed by adding a certain amount of ZIF-71. PVDF hollow fibre support membranes soaked in ethanol aqueous solution for 5 min and dried at room temperature and two ends sealed with silicone rubber. Membranes immersed in PVDF dilute solution for 10 s, and immediately immersed in pure water for 24 h.	0.7–1.2 µm sized particles 1 µm thick selective layer 0–2 wt% loading	VMD desalination Flux-27.1 kg/m^2^ h	[74]
16.	ZIF-8@RMs ZIF-8 on resin microspeheres	PolyphenylsulfonePPSU/ZIF-8@RMs	NMP	Phase inversion. PPSU dissolved in NMP and ZIF-8@RMs added into the solution. Ultrasonication for 30 min, stirring for 12 h at 25 °C and left overnight. Suspension was cast onto a clean rectangular steel plate at room temperature, immersed in a DI water coagulation bath for 15 min. Placed in freshwater for 24. Dried in air at room temperature overnight.	10 µm sized particles 100 µm thick membranes 5 wt% loading	Dye rejection from methanol solutions	[75]
17.	ZIF-L	Polyethersulfone (PES)PES/ZIF-L	NMP	Non-solvent induced phase separation method at room temperature. PVP powder and methanol-wetted ZIF-L nanoflakes mixed into NMP, ultrasonication for 10 min, followed by stirring for 30 min. PES added. Overnight stirring and degassing for 8 h. Membrane cast on a glass plate at room temperature and immersed in DI water for 24 h.	0.25, 0.5, 1 wt% loadings (0.5% optimum) Leaf-like nanoflakes with 150 nm thickness	Improvement of water flux	[76]
18.	ZIF-8	Polyethyleneglycol (PEG)	Water	ZIF-8 particles dispersion and PEG aqueous solution mixed and stirred. Maleic anhydride (crosslinking agent) and trimethylamine (catalyst) added with magnetic stirring. Solution cast on a PVDF supporting membrane and kept at room temperature for 12 h, followed by cross-linking for 5 h at 80 °C.	0, 2, 4, 6 wt% loading (optimum- 4 wt%)	Desulphurisation Thiophene/n-heptane mixture Flux-1960 g/m^2^ h Enrichment factor-8.93	[77]
19.	Carbonized ZIF-8 (CZIF-8)	Polyimide (PI) CZIF-8/PI	Water	Non-solvent induced phase separation (NIPS) method was used. PAA solution with CZIF-8 stirred at room temperature for 5 h, followed by sonication for 30 min and degassing in vacuum for 30 min. Solution cast on the PET non-woven fabric, immersed in DI water coagulation bath for 30 min, dried and thermally treated under nitrogen.	0–20 wt% loading (10 wt% optimum)	Nanofiltration performance; rejection of dyes (rose bengal, methyl blue, congo red, chrome black T) from aqueous and alcoholic (ethanol, isopropanol) solutions Approx-95% rejection of congo red from alcoholic solutions	[78]
20.	β-cyclodextrin-enhanced ZIF-8 (β-CD@ZIF-8)	poly(m-phenylene isophthalamide) (PMIA)	NA	The PMIA support layer formed onto the PI nanofiber (formed by electrospinning) by NIPS method and spin coating of PMIA with a spinner and IP between mphenylenediamine (MPD) and trimesoyl chloride (TMC) with the improvement of β-CD@ZIF-8.	87.1 ± 10.7 nm sized particles 102.1 ± 4.0 nm thick selective layer0.05 wt% loading	Organic solvent nanofiltration (dye/solvent mixtures)	[79]

## Data Availability

Not applicable.
